# Minimal residual disease in multiple myeloma: the movement towards response-adapted therapy

**DOI:** 10.3389/fimmu.2026.1932648

**Published:** 2026-09-25

**Authors:** Kenneth JC Lim, Holly Pertile, Andrew Spencer

**Affiliations:** 1 The Australian Centre for Blood Diseases, Monash University, Melbourne, VIC, Australia; 2 Department of Haematology, The Alfred Hospital, Melbourne, VIC, Australia; 3 Department of Haematology, The Northern Hospital, Epping, VIC, Australia; 4 Department of Haematology, St Vincent’s Hospital Melbourne, Fitzroy, VIC, Australia

**Keywords:** mass spectometry, MRD - measurable residual disease, myeloma, next generating sequencing, next generation flow cytometry (NGF), response adapted therapy, risk adapted management

## Abstract

In recent years, minimal residual disease (MRD) testing has emerged as a powerful prognostic tool in multiple myeloma. Multiple meta-analyses have demonstrated that achieving MRD negativity is associated with significantly improved progression-free survival (PFS), leading to its acceptance as a surrogate endpoint in clinical trials to support accelerated drug approval. Looking forward, MRD status is poised to be incorporated into treatment decision-making; to guide escalation and de-escalation of therapy with the aims of improved progression free survival with limited treatment toxicity. In this review article, we provide an overview of the current modalities available for MRD testing, including bone marrow, peripheral blood, and imaging-based techniques. We examine the prognostic significance of MRD status, with a focus on the impact of sustained MRD negativity, outcomes with MRD resurgence, and the depth of MRD negativity on progression free survival. Finally, we discuss the rapidly evolving landscape of MRD-adapted clinical trials and how these studies are informing the development of response-adapted treatment strategies.

## 1 Introduction

With the ever-increasing armamentarium of therapeutic options to treat Multiple Myeloma (MM), overall response rates (ORR) have exceeded 90% in both newly diagnosed transplant-eligible (NDTE) and newly diagnosed transplant-ineligible (NDTI) patients (1, 2). Complete response (CR) rates have also exceeded 80% and 50% in these two groups respectively. Similarly, in the relapsed/refractory (RR) setting, ORR have now exceeded 60% with the introduction of T-cell redirection therapy (3–5). This has translated to improved progression free survival (PFS) and overall survival (OS) in contemporary MM patient populations (6). This poses a problem, albeit a good one, for the conduct of clinical trials, given that OS, the “gold standard” of clinical trial endpoints, may no longer be practical due to the protracted timeframe for result readout. As such, the concept of minimal residual disease (MRD) status has been proposed as a surrogate endpoint. MRD negativity shows a strong association with PFS and a moderate association with OS in newly diagnosed (ND) patients (7). Since its conception, the application of MRD in MM now has clear definitions, thresholds, and accepted testing methods. However, the field continues to evolve with the improvement of MRD testing sensitivities and the development of peripheral blood techniques. *Post-hoc* analyses of many clinical trials have also demonstrated the impact of sustained MRD and MRD resurgence on survival, even amongst patients who had achieved a CR to treatment. A better understanding of the prognostic significance of MRD negativity (and positivity) has led to the development of multiple MRD-directed clinical trials, which has spearheaded the movement towards personalized therapy, with a risk-directed and response-adapted approach. This review article summarizes the current literature and future directions for MRD utilization in MM.

## 2 Definition of MRD negativity

In 2016, the International Myeloma Working Group (IMWG) expanded on its standard response criteria of stringent complete response (sCR), complete response (CR), very good partial response (VGPR), partial response (PR), minimal response (MR), stable disease (SD), and progressive disease (PD) to include MRD (8). In its criteria, the IMWG defines MRD negativity as the absence of clonal aberrant plasma cells at the level of 1 in 10^5^ (sensitivity of 10^-5^) normal bone marrow (BM) cells using multi-parameter flow cytometry (MFC) or next-generation sequencing (NGS) in patients with a CR. In addition, it included the use of imaging with negativity defined as the absence or disappearance of any positron emission tomography/computed tomography (PET/CT) avid lesions found at baseline to the level of less than the mediastinal blood pool standardized uptake value (SUV; ≤Deauville 2) or less than the surrounding normal tissue to complement the use of MRD. Since then, however, analyses of MRD data have loosely adapted the above definition. Some analyses have included patients with VGPR in their MRD-negative cohorts owing to reports showing that patients with negative MRD but persistent monoclonal protein (M-protein) have similar survival outcomes to those with undetectable MRD in CR (9, 10). Explanations provided for positive M-protein despite undetectable MRD include long immunoglobulin half-lives, oligoclonal reconstitution after treatment, interference by therapeutic monoclonal antibodies, and inability to confirm CR due to unavailable measurements in clinical trials. In the imaging arena, the definition of a complete metabolic response (CMR) has been subsequently revised to a threshold of ≤Deauville 3 (11).

## 3 Techniques for MRD testing

**Figure 1** provides an overview of techniques that can be used for MRD assessment.

**Figure 1:**
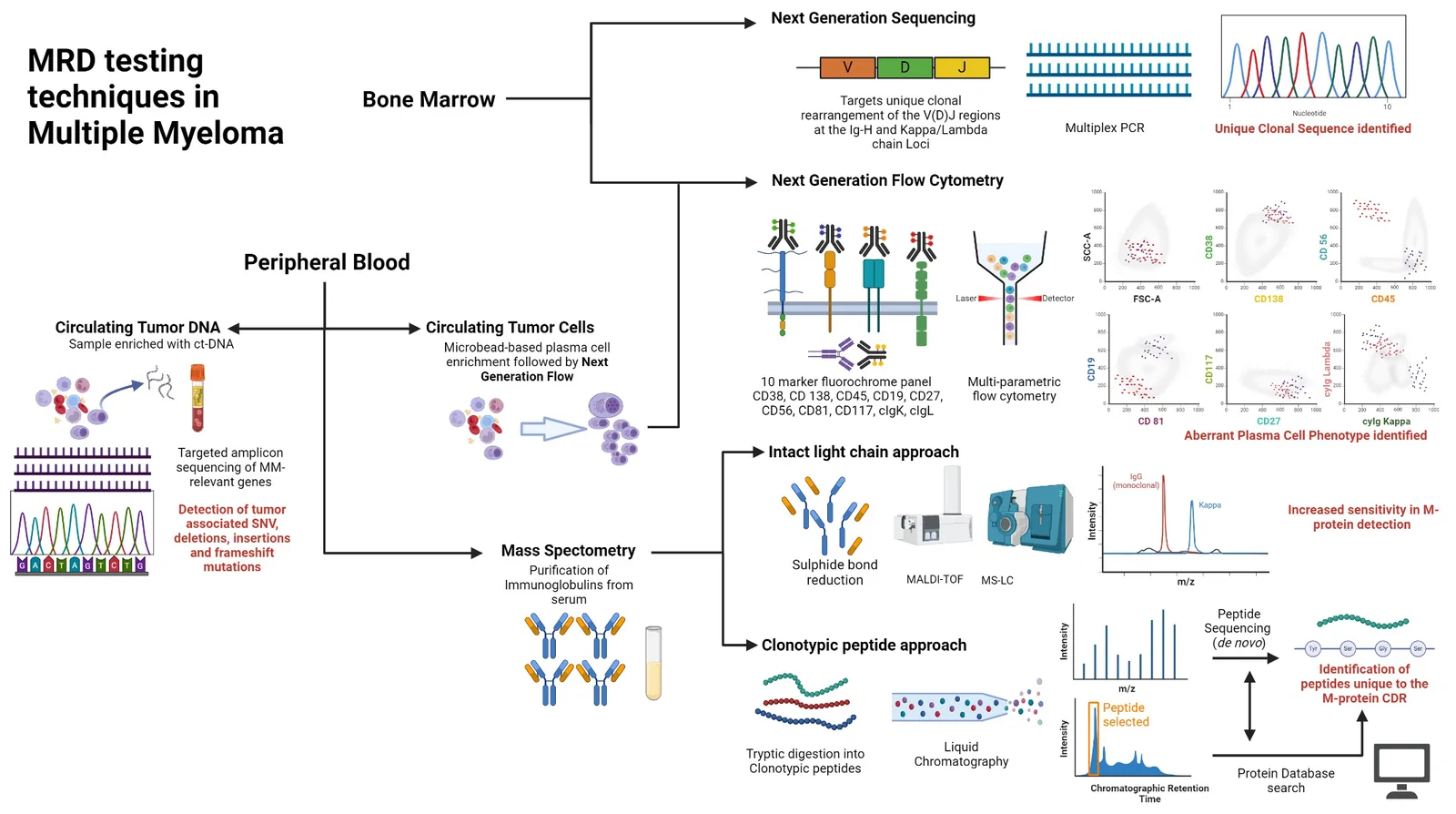
MRD testing techniques in multiple myeloma. Created with BioRender.com.

### 3.1 Established techniques

#### 3.1.1 Next generation flow cytometry

MFC utilizes the principle that MM plasma cells can be distinguished from normal plasma cells through immunophenotyping; the demonstration of restricted cytoplasmic κ or λ immunoglobulin light chain expression, differing expression patterns of known plasma cell surface markers CD138, CD38, CD45, CD56 and CD19 together with aberrant expression of other non-plasma cell markers such as CD27, CD28, CD81 and CD117 (12). The first generation of MFC methods used for MRD detection involved 4 or 6-color flow cytometry with a sensitivity of up to ≥1 in 10^4^ cells. Pioneering studies using these assays have subsequently demonstrated that MRD negativity translated to superior PFS and proved prognostic even amongst patients who achieved a CR to therapy (10, 13, 14). The development of the second-generation eight-colour MFC allowed for more robust interrogation of surface markers at one time, leading to increased sensitivity and specificity of the assay. To eliminate variability and inconsistency in MFC utilization amongst different testing laboratories, a standardized approach for MRD was developed by the EuroFlow Consortium now termed next generation flow cytometry (NGF) (15, 16). NGF involved careful selection of two eight-colour fluorochrome combinations which combined the analysis of 10 markers (CD38, CD138, CD45, CD19, CD27, CD56, CD81, CD117, cytoplasmic IgK and cytoplasmic IgL) (17). It also involved implementing strict procedures for instrument setup, fluorescence compensation, sample preparation, and developing an automated software tool to aid result evaluation. This method had an improved limit of detection (LoD) of 2×10^-6^ and a limit of quantification (LoQ) of 5×10^-6^, which has aided the standardization of analysis and reporting (17). NGF, however, does come with its own set of limitations. Aside from requiring at least 10 million cells for MRD measurement, conferring high dependency on BM sample quality, it requires processing and analysis within 48 hours of collection and relies on a high level of expertise to accurately identify a myeloma cell phenotype (8, 18).

#### 3.1.2 Next generation sequencing

Pioneered in Acute Lymphoblastic Lymphoma (ALL), molecular methods of MRD testing similarly target the hypervariable region of a myeloma cell (19, 20). These include the variable, diversity, and joining (VDJ) and the variable and joining (VJ) regions at the immunoglobulin heavy chain and kappa/lambda chain loci, respectively (21). In an older strategy using allele-specific oligonucleotide quantitative polymerase chain reaction (ASO-PCR), the unique clonal arrangement of the MM cell is first identified and sequenced (22). This is followed by the design of a unique combination of primers and probes used for amplification of the targeted oligonucleotide sequence by qPCR. Although this approach achieves a sensitivity threshold of <10^-5^, it is time-consuming, labour-intensive, and has shown variable applicability in MM. ASO-PCR has now largely been displaced by NGS.

NGS is another method approved by the IMWG for MRD assessment (8). Unlike ASO-PCR, a pre-made cocktail of locus-specific primer sets is used in NGS, allowing for the amplification of all VDJ re-arrangements present in the sample. Clonotypic sequences unique to the myeloma cell are identified if they comprise at least 5% (defined as monoclonal) amongst all the other polyclonal sequences. NGS is performed at the time of diagnosis and tracked at subsequent time points. Multiple commercial assays are available and in use, however the clonoSEQ^®^ assay (Adaptive Biotechnologies Corporation, Seattle, USA) is the only FDA-authorized assay (20). ClonoSEQ^®^ has a LoD of 6.77×10^–7^ and a LoQ of 1.76×10^–6^ at an maximum input of 20 μg of DNA (20, 23). Similarly to NGF, MRD negativity by NGS has been shown to be a strong prognosticator of survival outcomes (PFS, time to tumour progression (TTP), and OS) (21, 24). At the same sensitivity, NGS also has significant biological and clinical concordance with NGF and is used as an alternative to NGF (25, 26). The advantage of NGS over NGF is its lower limit of detection, with the ability to detect MRD at levels at 10^–6^ or 10^-7^ (23). NGS-based methods, however, are also highly dependent on BM sample quality to allow for adequate tumour DNA. In addition, a minority of cases (<10%) do not have an easily identifiable clone by NGS. It is also expensive and requires staff with a highly specialized skillset to run. As such, MRD by NGS is not as widely available and is mainly performed in centres with high sample throughput. **Table 1** provides a comparison of bone marrow MRD assessment using NGF and NGS.

**Table 1:** Comparison of bone marrow MRD assessment using next generation flow cytometry and next generation sequencing.

Test characteristics	NGF	NGS
Principle	Immunophenotyping of aberrant plasma cells	Sequencing of clonal rearrangements of the V(D)J regions of the IGH and kappa/ lambda chain loci
Standardization	Euroflow	ClonoSEQ is FDA-authorized Other commercial assays are available
Sensitivity	10^–6^ with an input of 10 million cells	10^–6^ with an input of 3 million cells
Baseline sample required	No	Yes, to identify trackable clone (NGS will fail to identify a trackable clone in 5-10% of patients and therefore cannot be used for MRD monitoring)
Ability to detect haemodilution	Yes	No, resulting in false negatives
Timing of sample handling	Must be processed within 24–48 hours of collection	Samples can be stored and processed at a later time
Turnaround	Hours	Days to weeks
Cost	Cheaper	More expensive

NGF, next generation flow cytometry; NGS, next generation sequencing; VDJ, variable, diversity and joining; IGH, immunoglobulin heavy chain; MRD, minimal residual disease.

### 3.2 Imaging modality and MRD

Due to the heterogenous, multi-focal nature of MM, disease assessment from a single BM aspirate might not be truly representative of the overall disease state particularly in the setting of extra-medullary disease. Hence, the use of 18F-FDG-PET/CT (PET/CT) is important as part of MRD assessment to ensure the complete eradication of detectable disease. Previously, PET/CT has been shown to have prognostic value; PET/CT positivity in NDMM patients who achieved conventional CR from therapy was associated with poorer survival outcomes (27, 28).

However, with the advent of MRD, a more sensitive method of detecting residual disease, one does wonder if there is any added value of imaging; is imaging able to detect residual disease in MRD-negative patients, and if so, are these imaging findings prognostically significant? Indeed, in a study comparing BM and imaging-based MRD assessment, 12% of patients who achieved BM MRD negativity by flow cytometry had a positive PET/CT or whole-body diffusion-weighted MRI (WB-DWI-MRI) and had a shorter PFS in comparison to patients with both BM and imaging MRD negativity (29).

The initial consensus of defining imaging response by PET/CT in association with MRD used the Deauville criteria, a five-point scoring system used to standardize the scoring of PET/CT images (30). It set the criteria of an imaging response to be a Deauville score of ≤ 2 (uptake ≤ mediastinum). Subsequently, the definition of CMR was standardized by Zamagni et al. to be all pre-existing focal lesions and BM FDG uptake to be a Deauville score of ≤ 3 (lower than the liver background) after proving negativity at this threshold to be an independent predictor of PFS and OS (31). Using this criterion, the CASSIOPET study showed that PFS was superior in patients who were baseline PET/CT-negative vs PET/CT-positive (32). More importantly, 11% of MRD-negative patients by NGF (10^-5^) were positive for PET/CT. Similarly, Moreau et al. also showed that 16% of MRD-negative patients (MFC 10^-4^) were still PET/CT positive, suggesting that the addition of PET/CT may “downgrade” the response of a small subset of otherwise MRD-negative patients (33). Interestingly, using a deeper threshold of MRD at 10^-6^ (NGS), Fonseca et al. found high rates of PET/CT negativity in MRD-positive patients, suggesting that increasing the sensitivity threshold of MRD detection may reduce the need for PET/CT (34).

Whole-body MRI (WB-MRI) is another imaging modality used in treatment response although its high cost may be prohibitive. In addition, the literature currently favours the use of PET/CT. In a study comparing the diagnostic efficacy of PET-CT vs WB-MRI in patients post Autologous stem cell transplant (ASCT), PET-CT was overall more accurate in determining remission status, albeit with a lower sensitivity but higher specificity (35). In a study by Moreau et al., PET-CT normalization after three cycles of bortezomib, lenalidomide and dexamethasone (VRd) was prognostic but MRI normalization was not (36).

### 3.3 Peripheral blood-based techniques

Peripheral blood (PB)-based methods are non-invasive tools used for disease monitoring and assessment of treatment response. Due to its invasive nature, more frequent BM sampling (i.e., monthly) may not be feasible for robust MRD monitoring. To obtain enough cellular events or DNA material, the “first pull” of the BM aspirate must also be used, an important pre-analytical variable. In addition, MM involves multi-focal sites, and a sample from a single BM site may not be fully representative of the spatial heterogeneous response to treatment in some patients. Collection and analysis of PB can be done more frequently and is not as affected by the irregular distribution of tumour cells in the body, potentially providing a more dynamic and accurate representation of disease response to treatment.

Several complementary PB-based strategies are under active investigation. Cell-free approaches, including mass spectrometry and sequencing of circulating tumour DNA (ctDNA), enable sensitive detection of tumour-derived analytes but do not preserve intact cells for downstream characterisation. In contrast, cell-based enrichment platforms, particularly immunomagnetic enrichment of CD138^+^ circulating plasma cells, permit isolation of viable intact tumour cells from PB, enabling not only ultrasensitive MRD detection when coupled with NGF or NGS, but also downstream genomic and clonotypic characterisation (37). This capacity for molecular profiling of circulating tumour cells is uniquely relevant to treatment re-escalation strategies, as it may reveal clonal evolution and resistance mechanisms that neither cell-free DNA nor mass spectrometry-based approaches can fully capture. The use of PB techniques is increasingly being explored and may in the future serve to complement rather than replace BM-based testing.

#### 3.3.1 Circulating tumour plasma cells

The concept of circulating plasma tumour cells (CTPC) detection uses NGF on the PB which allows for the detection of lower levels of circulating disease not otherwise detected by conventional MFC. Detectable CTPC has been shown to be a biomarker for adverse clinical outcomes and can be detected in the vast majority of newly diagnosed patients (38–40). Based on samples from NDMM patients enrolled in the GEM2012MENOS65 and GEM2014MAIN trials, 92% were CTPC detectable (40). Given the prevalence, a proposed cutoff of 0.01% CTPC was further shown to be an independent poor prognostic factor at diagnosis after accounting for ISS, LDH and cytogenetic factors (40). However, following treatment, CTPC remains a less sensitive MRD marker than BM MRD, with 40% of patients with detectable MRD in the BM negative for CTPC (41). To increase the sensitivity of NGF on the PB, Lasa et al. developed a technique termed “BloodFlow” (42). To achieve a LOD below 10^-6^, this technique involves the collection of larger volumes of peripheral blood (around 50mL) to allow for the analysis of ≥ 2×10^–8^ cells. Immunomagnetic enrichment is then used to isolate plasma cells into a smaller aliquot of 100μL prior to EuroFlow NGF. In an analysis of paired PB and BM samples analysed using BloodFlow and NGF respectively, the positive predictive value (PPV) and negative predictive value (NPV) of BloodFlow was 95.1% and 76.6% respectively. Moreover, among 156 patients (enrolled in the GEM2014MAIN and GEM2017FIT studies) undergoing maintenance or observation, patients with positive peripheral residual disease (PRD) by BloodFlow showed a 2-year PFS rates from last PRD assessment of 0% compared to 81% in patients with undetectable PRD respectively.

#### 3.3.2 Circulating tumour DNA

The principle of DNA-based MRD assessment of the BM can be translated to the PB through the analysis of circulating tumour DNA (ctDNA), which has been shown to mirror the genomic profile of the bone marrow clone (43, 44). Methods of ctDNA assessment include the use of droplet digital PCR (ddPCR), targeted deep sequencing panels and whole-exome (WES) or whole-genome (WGS) sequencing to identify genetic alterations of the tumour clone (43, 45, 46). In addition to tracking genomic abnormalities, tumour-specific immunoglobulin rearrangements can be sequenced and monitored (47). However, current ctDNA methods have failed to replicate the sensitivity of BM-based techniques. Using a tumour-informed approach, where the baseline genomic profile of the tumour clone is established and then tracked, is one method of increasing the sensitivity of ctDNA based monitoring (48, 49). Buenache et al. have recently employed a technique known as AltumTrackSeq, which utilizes larger volumes of blood (50mL; to increase DNA input) for analysis with tumour-informed amplicon-multiplexed mini-panels paired with tandem single-nucleotide variant sequencing (tSNV-Seq) to improve the sensitivity of ctDNA analysis on the PB (up to 10^-6^). In their analysis of 84 ND patients, compared to BM NGF assessment, their ctDNA approach showed a greater PPV (88.9% vs. 34.5%) and specificity (98.4% vs. 70.3%) for predicting relapse, however failed to achieve the sensitivity of BM NGF (61.5% vs. 83.3%). Additionally, they demonstrated that in combination with BM-based MRD positivity, ctDNA positivity compared to ctDNA negativity conferred an inferior prognosis, indicating ctDNA may aid to improve risk-stratification of patients in addition to BM MRD monitoring. This is in line with findings from previous studies (50, 51).

In addition, there may be a role for ctDNA in monitoring patients with oligosecretory, non-secretory, and extra-medullary (EM) MM (52, 53). Currently, imaging-based techniques (PET/CT) and serial BM assessments are the only ways to monitor disease response in this group of patients. In a study using time-matched EM plasmacytoma biopsies, BM aspirates, and PB from 8 patients, there was a higher concordance of PB ctDNA to tissue biopsy (0.873) compared to bone marrow aspirate (BMA) to tissue biopsy (0.621) (54). This suggests that ctDNA may allow us to more accurately and frequently measure disease burden in patients with historically “non-measurable” disease.

### 3.4 Mass spectrometry in peripheral blood

Detection of the M-protein is conventionally done through the combination of serum protein electrophoresis (SPEP) and immunofixation (IFE). Historically, these techniques have a LoD of 1g/L and 0.5g/L, respectively. Newer mass spectrometry (MS)-based techniques, however, have demonstrated the ability to detect this protein at a much lower concentration with the added ability to differentiate the M-protein from interfering therapeutic monoclonal and bispecific antibodies (daratumumab, isatuximab, teclistamab, elranatamab and talquetemab etc.) used in MM. The premise of using MS to measure MM is based on the concept that each myeloma cell clone contains unique heavy and light chain genes which in turn produce a unique immunoglobulin (M-protein) with a unique amino acid sequence at the antigen binding region or complementarity determining region (CDR) resulting in a different overall mass to charge ratio (55). As MS requires a measurable secretory clone, this approach cannot be used for patients with non-secretory disease. Two methods have been described in the literature, both of which involve sample enrichment for immunoglobulins prior to analysis by MS.

#### 3.4.1 Intact immunoglobulin light chain approach

In the first approach (intact immunoglobulin light chain approach), a sample enriched with intact monoclonal protein is introduced into the mass spectrometer and then analysed. The MASS-FIX assay has been extensively validated and currently used for clinical purposes at the Mayo Clinic (56). It involves enriching a patient sample through isotype-specific (IgG, IgA, IgM, IgL and IgK) camelid-derived nanobodies linked to agarose beads and separation into 5 isotype-specific aliquots. The isolated immunoglobulins are then dissociated into heavy and light chains and then run through the matrix-assisted laser desorption/ionization, time of flight mass spectrometer (MALDI-TOF MS) (56, 57). The MALDI-TOF MS ionizes samples into the gas phase and measures and plots their mass-to-charge (m/z) ratio according to their time of flight through the flight tube (57). The EXENT^®^ (The Binding Site) assay utilizes a different immuno-enrichment technique involving polyclonal (containing IgG, IgA, IgM, IgL, and IgK) sheep nanobodies covalently linked to paramagnetic beads. The bound proteins are then eluted off, dissociated, and analysed by MALDI-TOF MS (58). Both MALDI-TOF MS methods allow for the identification of M-protein at a sensitivity <0.1g/L compared to conventional SPEP and IFE. A third assay, referred to as Liquid chromatography-mass spectrometry (LC-MS), uses similar immuno-enrichment methods but adds an additional liquid chromatography step to separate out the immunoglobulin light chains before ionization and passage through a mass spectrometer (59). This extra-step resulted in higher sensitivity of 0.001g/L.

#### 3.4.2 Clonotypic peptide approach

The second approach is called the clonotypic peptide approach, which represents the most sensitive method with a LoD of 0.001g/L due to the removal of interference from the polyclonal background (60). This approach is based on the principle that when digested by an enzyme such as trypsin, the CDR of an M-protein would break down into unique clonotypic peptides with unique fragmentation patterns. This technique requires a diagnostic sample for initial clonotypic peptide identification, usually obtained from the monoclonal band in the diagnostic SPEP. After immunoglobulin enrichment, the sample is digested into smaller peptides before analysis, separated by size and charge using LC before analysis by MS. Given that the CDR may contain framework/constant regions, the selection of clonotypic peptides for MRD monitoring must be carefully performed. This requires further genetic sequencing of the CDR from the malignant clone and referencing to a human proteome library to ensure uniqueness, usually with the help of a bioinformatics algorithm (23, 61, 62).

#### 3.4.3 Mass spectrometry compared with bone marrow MRD assays

MS-based approaches have emerged as promising complements, rather than replacements, to established BM MRD assays and their relative performances varies considerably by platform. MALDI-TOF MS methods demonstrate sensitivity broadly inferior to BM NGF and BM NGS, failing to detect a circulating paraprotein in >30% of BM NGF or BM NGS positive patients in the two separate clinical trials (63, 64). In contrast, the more sensitive, LC-MS and clonotypic peptide sequencing approaches, have been able to detect monoclonal protein in the majority (>70%) of patients classified as MRD-negative by established BM standards (65, 66). Whether these highly sensitive methods of detection, translate to true survival benefit, remains to be seen. Critically, the prognostic value of MS appears most powerful not in isolation but through integration with other BM or PB-based techniques. A recent analysis of paired BM and blood samples collected from the ATLAS trial found an association of double negativity on MALDI-TOF MS and BM NGF with superior PFS compared to MRD negativity by only 1 modality. The benefit of combinatorial assessment is particularly evident in relapsed and refractory disease and following novel immunotherapies such as CAR T-cell therapy and bispecific antibodies, settings where extramedullary disease may confound BM-centric evaluation (67).

#### 3.4.4 M-protein persistence and resurgence on mass spectrometry

One of the issues facing MS based methods is that low levels or circulating paraproteins may persist even after eradication of its producing plasma cell. The increased sensitivity of M-protein detection by MS has added complexity to the interpretation of BM-MS discordance, as it remains unclear whether MS positivity in the setting of BM MRD negativity represents biologically meaningful residual disease or a result of prolonged paraprotein clearance. In addition, the clearance rate of the M-protein depends heavily on its molecular size, structural valency and catabolic handling. For example, IgG has serum half-life of ~21 days compared to IgA’s ~6 days which translates to different speed of response for each isotype of immunoglobulin (68). Using increasingly sensitive methods to detect lower levels of M-protein, IgG in particular, may result in a long-time lag between MRD negativity in the BM and negativity by MS (69). It has also been well documented that a large proportion of patients with detectable M-protein by conventional methods and negative MRD would achieve CR later in time (70, 71). The clinical significance of assessment timing is well illustrated by the ATLAS trial, in which concordance between BM MRD and MS status was maximal only at 18 months post-treatment, with more than half of patients who converted from MS-positive to MS-negative doing so beyond the 12-month mark (72). This temporal disconnect likely reflects delayed clearance of circulating monoclonal protein rather than persistent active disease, and highlights an important pre-analytical consideration when interpreting early MS results. Contrarily, MS persistence, must be differentiated with MS resurgence, where MS resurgence may be attached to an impending or future relapse (63). Consequently, characterizing the kinetics of MS conversion relative to BM MRD negativity and understanding the prognostic significance of the trajectory and rate of change in MS readings over time represents an important avenue for future investigation.

## 4 MRD and its impact on survival

### 4.1 MRD negativity is associated with improved survival outcomes

Numerous meta-analyses have been performed to correlate MRD-negativity rates in clinical trials, as defined by the 2016 IMWG response criteria, to PFS and to a smaller extent OS. One of the first and largest analysis by Munshi et al. included 44 studies with ~8000 patients for PFS data and 23 studies with ~4000 patients for OS data (73). The analysis included NDTE, NDTI, and RR patients from trials with MRD testing done at variable time points (at VGPR, at CR, pre- and post-maintenance therapy), using different MRD sensitivity thresholds (10^-4^, 10^-5^,10^-6^) and MRD assessment methods (MFC, NGS, NGF). The investigators found that MRD negativity was associated with a significant reduction in progression (HR 0.33) and death (HR 0.45) when compared to MRD positivity. This is irrespective of disease setting, MRD testing method, sensitivity threshold, timing of MRD assessment, and cytogenetic risk.

### 4.2 MRD negativity as a surrogate endpoint

In 2024, in a unanimous decision of 12 to 0, the FDA’s Oncologic Drugs Advisory Committee (ODAC) approved MRD as an endpoint for accelerated approval of new therapies in MM; however, stopped short in calling for the replacement of PFS with an MRD-based surrogate endpoint, given the high-bar set for true surrogacy. Changes induced by a therapy on a surrogate endpoint should independently influence changes in a clinically meaningful endpoint such as PFS or OS. A few trial-level meta-analyses have been previously performed showing a moderate to strong correlation between MRD negativity and PFS (R^2^ 0.62-0.97); however, they only included trials in ND patients and contained studies using less sensitive MRD thresholds (>1.0×10^-5^) (24, 74). A more recent meta-analysis containing contemporary treatment regimens by Paiva et al. included 15 pivotal clinical trials with results published between 2016 – 2022 (75). Only studies with >50 patients per treatment arm and using NGF or NGS methods with a minimum sensitivity threshold of 10^–5^ were included. Studies included 6 trials of NDTE, 3 of NDTI, and 6 of RRMM patients. In their analysis, the treatment effect on MRD-negative rates moderately correlated with the treatment effect on PFS (R^2^ 0.70), and this, too, held in the NDTE, NDTI, and RR subgroups. Another analysis by Pavia et al. included 20 randomized clinical trials (9 in NDTE, 5 in NDTI and 6 in RRMM) and stratified these into tertiles based off the difference in MRD negativity rates observed in the experimental vs. control arms. The median PFS for the lower, middle, and upper tertiles was 3.2, 5.5 and 17.1 months respectively. The upper tertile included studies where MRD negativity rates differed by greater than 13.5%, and this difference was proposed as a reasonable endpoint for the predication of a significantly improved PFS in randomized clinical trials (76).

However, there remain limitations in the use of MRD as a surrogate endpoint. MRD negativity weighs heavily towards immediate treatment efficacy but fails to capture long-term treatment toxicity and other post-progression events. Other weaknesses in the meta-analyses performed include a lack of standardization of assays across trials, and inter-trial variability in design, follow-up and evaluation times, and baseline characteristics. There is also a paucity of data involving newer therapies such as monoclonal antibodies, small molecules, and T-cell redirection therapies.

As such, in January 2026, the FDA released a draft guideline outlining several key recommendations for use of MRD as a surrogate endpoint in clinical trials (77). Here, both MRD and CR are specified as acceptable as endpoints for accelerated drug approval in MM. Importantly, these cannot be used in all disease settings, as there is currently insufficient data to support MRD/CR as a surrogate endpoint in smouldering MM, MGUS or extramedullary disease, as well as in the maintenance setting. If using MRD as a surrogate endpoint, this must be assessed at a sensitivity of at least 1 in 100,000 (10^5^) residual tumour cells and the assay used must have a sensitivity at least one log below this threshold. Finally, post-approval confirmation of clinical benefit remains mandatory and must be underway before accelerated approval is granted. Products approved on MRD or CR endpoints must verify actual clinical benefit, for example improved PFS or OS, through confirmatory trials.

## 5 The impact of MRD at different time points

### 5.1 MRD testing time points

Unlike “curable” haematological malignancies such as Acute Leukaemias, where most therapy given is finite, MM treatment is usually administered continuously until the next progression (except Chimeric Antigen Receptor T-cell (CAR-T) therapy). In addition, the median time to best treatment response also varies depending on the therapeutic used. In the case of NDTE patients, treatment response rates improve after each phase of treatment (induction, ASCT, pre-maintenance, and maintenance). In the NDTI and RR setting, the median time to best response may range from 2 to 12 months, with some patients achieving their best response >12 months from treatment initiation (2–4, 78). While disease assessments performed using the IMWG response criteria (sCR, CR, VGPR, PR, SD, MR, and PD) involve non-invasive testing, namely urine SPEP, plasma SPEP and serum free light chains (SFLC), current MRD techniques require a BM aspirate and trephine biopsy, an invasive procedure. As such, to avoid excessive repeated testing, it is important to carefully select appropriate MRD assessment landmarks at different time points, which can provide important prognostic information to allow for treatment decision-making (23). This has proven challenging given the current heterogeneity of MRD assessment landmarks amongst clinical trials (73). Commonly used landmarks in the NDTE setting include post-induction, post-ASCT/pre-maintenance and at regular intervals (6–12 monthly) during maintenance therapy with MRD negativity status at the 1, 2, 3 year mark of particular interest (73). In the NDTI and RR setting, commonly used landmarks include at time of VGPR or CR and after a pre-specified treatment cycle or timepoint (i.e after 8 cycles or at 12 months).

### 5.2 MRD negativity post-induction, post ASCT, and pre/post-maintenance therapy is associated with better outcomes

It has been established that with modern day therapies for NDTE MM patients, rates of MRD negativity increase after each phase of treatment (1, 10, 79–81). In the age of quadruplet therapy, MRD negative rates (10^-5^) increase from around 40% post induction to 60% post ASCT/consolidation and approach 80% with maintenance therapy or post extended consolidation (1, 79, 82). Importantly, MRD negativity achieved post-induction and pre-maintenance is associated with superior PFS and OS, independent of the induction regimen used (81, 83, 84). In a *post-hoc* analyses of the GEM2012MENOS65/GEM2014MAIN studies, it was found that failure to achieve post-induction MRD negativity was associated with increased risk of MRD resurgence and/or PD (85). Similarly in a separate analysis of the FORTE study, patients who achieved MRD negativity earlier, pre-consolidation, had a lower risk of MRD resurgence than patients who achieved MRD negativity post-consolidation (86). In the IFM 2009 study involving patients receiving VRd with or without ASCT, patients who achieved MRD negativity (10^-6^) prior to initiation of 1 year maintenance therapy with lenalidomide had a 78% lower risk of progression or death compared to their MRD positive counterparts (median PFS not reached vs. 29 months) irrespective of whether patients received ASCT (83).

### 5.3 Resurgence of MRD may predict clinical relapse and is associated with poorer outcomes

It has been increasingly evident that there are a group of patients who achieve MRD negativity but fail to sustain it. These patients do as poorly as patients who never achieve MRD negativity. A retrospective study by Mohan et al. looked at patients who achieved post-ASCT MRD negativity (NGF 10^-5^) receiving immunomodulatory drug (IMiD) and proteosome inhibitor (PI) maintenance (87). Whilst the majority of patients had sustained MRD negativity (median follow up 10 years), 39% of MRD negative patients developed MRD resurgence, mostly within the first 5 years of achieving MRD negativity. Of these, about half developed a clinical relapse within a year.

In the Tourmaline MM3/MM4 study of NDTE patients, ixazomib was used as maintenance and compared to observation (88). MRD (MFC 4 × 10^-6^) assessment was performed at 0, 14 and 24 months from starting maintenance therapy. At the 14-month landmark analysis, previous MRD negative patients who developed MRD resurgence had a 2-year PFS of 34% compared to 75% in patients with sustained MRD negativity. Importantly, PFS in those with MRD resurgence was comparable to patients who were MRD positive pre-maintenance (2-year PFS 34% vs 28%). Of note, half of the cases of MRD resurgence occurred within 2 years of achieving MRD negativity.

### 5.4 Predictors of MRD resurgence

**Table 2** highlights key studies reporting predictors and outcomes following MRD resurgence. Aside from the aforementioned factor of longer time to achieving MRD negativity, other important predictors of MRD resurgence or progressive disease include high-risk disease by International Staging System (ISS) scoring, high-risk cytogenetic abnormalities (HRCA) namely amplification of 1q and ≥2 HRCA, and high circulating tumour cells at baseline (85, 86). In a cohort of patients receiving ASCT, MRD negative rates post-ASCT were similar amongst the 0, 1 or ≥2 HRCA subgroups. However, patients with ≥2 HRCA who initially achieved MRD negativity experienced a 41% cumulative resurgence of MRD within one year (92).

**Table 2:** Outcomes and predictors of MRD resurgence reported in key studies.

Study and population	Start of observation period	Follow up	Percentage of patients with MRD resurgence	Outcome following resurgence	Key points
Mohan et al. (87) NDTE	From diagnosis	Median follow up of 9.9 years	224/568 (39%)	Median time of 12 months from resurgence to clinical relapse	Highest risk period for resurgence was within 5 years of diagnosis; in a landmark analysis, MRD resurgence within 3 years of ASCT was associated with inferior PFS and OS compared with sustained MRD negativity
TOURMALINE-MM3 and MM4 trials (88) NDTE and NDTI	From start of maintenance	Median follow up for overall cohort not reported	111/229 (48.5%)	Median PFS of 12.4 months from the 14-month landmark (for those who had experienced resurgence by this timepoint)	Nearly half of MRD- patients had resurgence in <2 years
Terpos et al. (89) NDTE	From cessation of maintenance; Required 3 years of sustained MRD- before maintenance cessation	Median follow up of 3 years	12/52 (23%)	3-year PFS 80%	Half of the patients with resurgence (6/12) had ≤1 HRCA
MRD2STOP (90) NDTE and NDTI	From cessation of maintenance; required MRD- CR after ≤1 year of maintenance to cease	Median follow up of 30 months	11/45 (24%); 5 with PD	Not reported	Deeper MRD- at baseline (<10^-7^ vs. ≤10^-7^) was associated with a significantly greater MRD-free survival
GEM2014MAIN (91) NDTE	From start of maintenance	24 months	16/226 (7%)*	Median PFS of 10 months from the 24-month landmark^#^	Conversion of MRD+ to MRD- had a similar PFS to those with sustained MRD- from baseline; those with MRD resurgence had a worse prognosis
FORTE (86) NDTE	From first MRD- prior to or during maintenance	Median follow up of 50.4 months	118/306 (39%); 48 with PD	Median PFS of 22.3 months (for 70 patients with MRD resurgence before PD)	Amp(1q), ≥2 HRCA, high CTC, and a longer time to reach first MRD negativity were significant predictors of unsustained MRD negativity
MASTER (82) NDTE	From cessation of treatment; required MRD- on 2 consecutive assessments performed at prespecified timepoints from diagnosis to cease	Median follow up of 32.7 months	23/84 (27%); 16 with PD	Not reported	Risk of resurgence was significantly higher in patients with ≤2 HRCA

MRD, minimal residual disease; NDTE, newly diagnosed transplant eligible; PFS, progression-free survival; ASCT, autologous stem cell transplant; OS, overall survival; NDTI, newly diagnosed transplant ineligible; HRCA, high-risk cytogenetic abnormalities; PD, progressive disease; CTC, circulating tumour cells.

*Percentage of all patients included in study with MRD sample at 24 months.

^#^Applies specifically to patients negative at baseline and 1 year who converted positive at 2 years; narrower than the full 16/226 group.

### 5.5 Sustained MRD is associated with superior survival outcomes

As such, sustained MRD based on serial testing over a treatment course is increasingly emphasized as more important than MRD at any one timepoint in predicting the risk of disease progression. Defined by the IMWG, sustained MRD negativity refers to MRD negativity in the marrow and imaging performed at least 1 year apart (8). *Post-hoc* analysis of pivotal clinical trials have demonstrated that sustained MRD negativity is an excellent prognostic marker, independent of therapy arm (**Table 3**). In the IFM 2009 study which used a 1-year fixed duration of lenalidomide maintenance after VRd with or without ASCT, landmark analysis from the time of completion of maintenance therapy at 1 year sustained MRD negativity was associated with a 82% reduction in the hazard of progression or death compared with MRD-positive patients (83). What about MRD negativity past the 1-year mark? In the CASSOPEIA trial, the risk of progression or death was 80% and 92% lower in those who achieved 1 year and 2 year sustained MRD negativity respectively (80, 95, 96). A single-centre study by Diamond et al. of patients who received lenalidomide maintenance (until progression) also showed that no patients who achieved sustained MRD negativity (10^–5^ NGF) for 2 years developed disease progression (median follow-up of 19.8 months from the 2-year maintenance landmark analysis) (93). The FORTE study which randomized patient to carfilzomib and lenalidomide (KR) vs lenalidomide maintenance, showed that regardless of treatment arm, 4 year PFS was >96% if patients had 2 year sustained MRD by either NGF or NGS (26). Given that the sustained MRD negativity duration adds to the PFS measurement, the true value of sustained MRD negativity should be reflected in improved OS.

**Table 3:** Outcomes with sustained MRD negativity reported in key studies.

Study and population	Duration and timepoint of sustained MRD negativity	MRD method and sensitivity	Outcome
CASSIOPEIA (80) NDTE	1 year from post induction	MFC at 10^–5^ sensitivity	HR for PFS (sustained vs. unsustained) 0.20 (P<0.0001)
	2 years from post induction		HR for PFS (sustained vs. unsustained) 0.08 (P<0.0001)
Diamond et al. (93) NDTE and NDTI	2 years from start of maintenance	MFC at 10^–5^ sensitivity	100% PFS at a median follow-up of 19.8 months from the 2-year landmark
FORTE (26) NDTE	1 year from pre-maintenance MRD assessment	MFC at 10^–5^ sensitivity, NGF at 2.5×10^–6^ sensitivity and NGS at 10^–5^ and 10^–6^ sensitivity	4-year PFS from first randomization (prior to induction) 91% by MFC and 97% by NGS
	2 years from pre-maintenance MRD assessment		4-year PFS from first randomization (prior to induction) 99% by MFC and 97% by NGS
Liu et al. (94) NDTE	<1 year at any time during treatment*	MFC at 2×10^–5^ to 10^–5^ sensitivity	Median PFS 20.90 months from time of first MRD negativity
	1–2 years at any time during treatment		Median PFS 25.66 months from time of first MRD negativity
	2–3 years at any time during treatment		Median PFS 42.48 months from time of first MRD negativity
	3–4 years at any time during treatment		Median PFS 71.89 months from time of first MRD negativity
	≥ 4 years at any time during treatment		Median PFS 84.53 months from time of first MRD negativity
GEM2014MAIN (91) NDTE	1 year (MRD+ at the start of maintenance but MRD- at assessment after 1 year of maintenance and at the 2-year landmark)	NGF at 2×10^–6^ sensitivity	4-year PFS 83% from the landmark at 2 years of maintenance therapy^#^
	2 years from start of maintenance		4-year PFS 86% from the landmark at 2 years of maintenance therapy
MASTER (82) NDTE	≥ 12 months at any time during treatment	NGS at 10^–5^ sensitivity	36-month PFS 89% from the landmark at 18 months from enrollment
TOURMALINE-MM3 and MM4 trials (88) NDTE and NDTI	14 months from start of maintenance	MFC at 4×10^–6^ sensitivity	Median PFS 26.5 months from the 14-month landmark 2-year PFS 75.0% from the 14-month landmark
	28 months from start of maintenance		Median PFS NR from the 28-month landmark 1-year PFS of 96.6% from the 28-month landmark

MRD, minimal residual disease; NDTE, newly diagnosed transplant eligible; MFC, multiparameter flow cytometry; HR, hazard ratio; PFS, progression-free survival; NDTI, newly diagnosed transplant ineligible; NGF, next generation flow cytometry; NGS, next generation sequencing; NR, not reached.

*Patients must have had 2 consecutive MRD negative results and available cytogenetics data to be included in study.

^#^Maintenance was discontinued at this 2-year landmark if MRD negative.

## 6 Impact of the depth of MRD

It has been increasingly evident that depth of response does matter and that significant reduction in MM clone size reduces the incidence of clonal evolution and selection for treatment-resistant clones (97). The meta-analysis by Munshi et al. established that the risk of progression/death falls with each log-reduction of MRD detection threshold with a threshold of 10^–6^ having the highest discriminatory effect on PFS (vs 10^–4^ and 10^–5^) (73). *Post-hoc* analysis of the MASTER and IFM 2009 trials also demonstrated superior PFS in patients who reached MRD < 10^–6^ compared to patients with MRD between 10^–5^ and 10^–6^ (83, 98).

The MRD2STOP trial tests the concept of using NGS based MRD at a lower threshold of 10^–6^ to guide the discontinuation of maintenance therapy (23, 90). After a minimum of 1 year of maintenance therapy, participants could discontinue treatment if they achieved MRD negativity by NGS at the sensitivity of 10^–6^, NGF at 10^–5^ and PET/CT. Whilst data showed that 3 year PFS was 85% after treatment discontinuation, an exploratory analysis using immunomagnetic enrichment for a more sensitive MRD threshold at 10^–7^ showed that 3 year PFS of MRD positive cases was 49% vs 92% in MRD negative ones (23, 90).

However, the quest for deeper MRD responses should not come at the expense of excessive treatment toxicity, particularly when achieving MRD negativity at the 10^–5^ threshold may be sufficient for the majority of MM patients to remain progression free for a protracted period of time. A group that may have a greater benefit to risk ratio for achieving a deeper MRD response, however, are those with high risk MM. MM with HRCA undergo significantly higher rates of genomic changes over time compared to standard-risk disease (97). It is also evident that MRD negativity at <10^–5^ does not sustain in a large proportion of ultra-high risk disease, particular patients with amp1q or ≥2 HRCA (82, 85, 86, 98, 99). As such, achieving deeper MRD responses may reduce the risk of clonal selection and evolution which may translate to more sustained treatment effects. Currently, two studies investigating the use of quadruple/quintuplet therapy in high-risk MM have reported early MRD data with MRD negative rates of >60% at the threshold of 10^–5^ (99, 100). It would be interesting to observe outcomes of patients achieving MRD negativity at the level of <10^–6^.

Conversely, it is also important to recognize the concept of MM with MGUS-like phenotype, a subgroup of patients with low rates of disease progression even with measurable disease post treatment (101). In a study by Burgos et al. who used an algorithm based on percentages of BM plasma cells and of clonal plasma cells in the BM plasma cell compartment, MM patients with MGUS-like phenotype showed PFS and OS rates of 79% and 96% at 5 years with the achievement of CR or MRD negativity having no impact on PFS or OS (101).

## 7 Practical aspects of MRD: can MRD be used to guide decision making?

**Figure 2** summarizes clinical trials investigating the role of MRD-guided treatment decision making. **Boxes 1**-**3** highlights key practice points on MRD-guided decision making.

**Figure 2:**
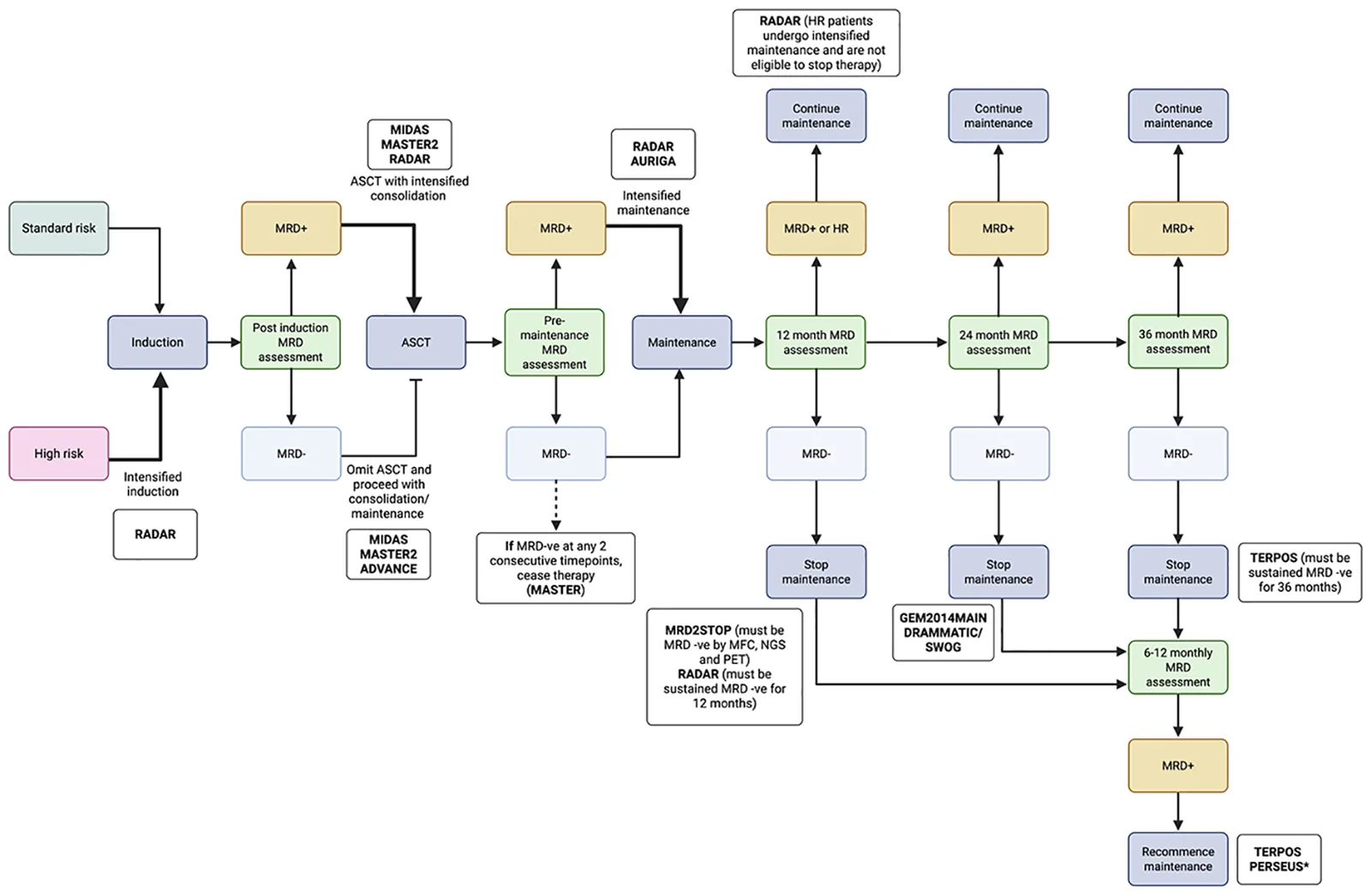
MRD-guided treatment decision making in newly diagnosed transplant eligible myeloma: recommendations based on clinical trials. Created with BioRender.com. *In PERSEUS patients randomized to receive Dara-R maintenance could stop Dara after 24 months of maintenance if 12 months sustained MRD-. This was restarted on resurgence.

Box 1 **Therapy can be ceased or de-escalated in patients with sustained MRD negativity.**

- More sensitive MRD thresholds (10^-6^) may be used to stop therapy.
- Sustained MRD negativity may be used to stop therapy.
- Patients with sustained MRD negativity may not benefit from prolonged maintenance therapy with daratumumab, lenalidomide or ixazomib.
- A randomized controlled trial is in progress that will compare stopping vs. continuing lenalidomide maintenance after 2 years of sustained MRD negativity.

Box 2 **Therapy cessation or de-escalation based on MM risk profile.**

- Sustained MRD negativity may be a viable endpoint for treatment cessation in standard risk patients but not high-risk ones.

Box 3 **Clinical trials on MRD directed treatment intensification are ongoing.**

- MRD may help guide decisions on the use of upfront ASCT, consolidation and multi-drug maintenance in the future.
- The role of treatment re-escalation in MRD resurgence is currently being studied.

### 7.1 Therapy can be ceased or de-escalated in patients with sustained MRD negativity

Based on data from the aforementioned IFM 2009 and MRD2STOP studies, MRD negativity (at 10^-6^) after at least one year of fixed duration lenalidomide maintenance therapy demonstrates that >75% of patients will remain progression free at three years from treatment cessation (83, 90). The Myeloma XI clinical trial, which randomized patients to lenalidomide maintenance vs observation post-ASCT showed in their landmark analysis at three years, patients who remained MRD negative (MFC 4 ×10^–5^) at did not benefit as much from continued lenalidomide maintenance vs observation (102, 103). In the CASSIOPEIA trial, patients who achieved sustained CR with MRD negativity after daratumumab, bortezomib, thalidomide and dexamethasone (Dara-VTd) induction, ASCT, and Dara-VTd consolidation did not benefit from the addition of daratumumab maintenance vs observation alone (80). In the Tourmaline MM3 and MM4 studies investigating the use of ixazomib maintenance vs observation, ixazomib maintenance only improved PFS in patients who were MRD-positive pre-maintenance (88). All these suggest that the incremental progression free survival benefit from indefinite maintenance therapy may be modest in patients with sustained MRD negativity. Conversely, the GEM2014MAIN trial questioned the benefit of continued lenalidomide based maintenance at 2 years if patients remain MRD positive. In this study, patients were able to cease maintenance (ixazomib, lenalidomide and dexamethasone or lenalidomide and dexamethasone) if they were MRD negative after 24 months of maintenance. MRD positive patients at this timepoint continued with lenalidomide and dexamethasone for a further 3 years. The study found that the 4-year PFS from the decision to cease therapy was 82.8% for those with MRD negativity and treatment cessation vs. only 50.4% with MRD positivity and treatment continuation (91).

More importantly, there will finally be some prospective randomized controlled trial data investigating the benefit of prolonged lenalidomide maintenance vs. observation in patients with sustained MRD negativity. In the DRAMMATIC (SWOG S1803) study, NDTE patients receiving either maintenance daratumumab and lenalidomide or single agent lenalidomide post-ASCT will undergo a second randomization to continue or stop maintenance therapy if they remain MRD negative (<10^–5^) after two years (104).

### 7.2 Therapy cessation or de-escalation based on MM risk profile

In the phase III ATLAS trial comparing carfilzomib, lenalidomide and dexamethasone (KRd) versus lenalidomide maintenance, standard risk patients in the KRd arm were de-escalated to lenalidomide if they achieved MRD negativity (< 10^–5^) after 6 cycles (105). Standard risk patients who underwent early de-escalation had similar outcomes compared to patients who received all 36 cycles of KRd. This suggests that prolonged intensive treatment may not benefit standard risk patients who achieve early MRD negativity (106). Conversely, in the phase 2 MASTER trial utilizing daratumumab, carfilzomib, lenalidomide and dexamethasone (Dara-KRd) induction, ASCT followed by Dara-KRd consolidation allowed cessation of therapy if MRD was achieved at 2 consecutive predefined timepoints (82). Patients with 0–1 HRCA who had stopped therapy had a 2 year PFS of >85% compared to 53% in patients with ≥2 HRCA.

### 7.3 Clinical trials on MRD directed treatment intensification are ongoing

What if patients remain MRD positive or develop MRD resurgence? Is there a role for treatment intensification? The AURIGA study compared intensification of maintenance with MRD positive patients randomized to daratumumab and lenalidomide (Dara-R) vs. lenalidomide maintenance (107). At a median follow up of 32.3 months, the rate of conversion to MRD negativity was 60.6% in the Dara-R group compared to 27.7% in the lenalidomide group (OR 4.12, 95% CI 2.26-7.52, P<0.0001). Furthermore, the rate of sustained MRD negativity ≥6 months for Dara-R was 2.5 times that of lenalidomide alone. The MRD-driven consolidation phase of MIDAS study compared escalation of consolidation in patients who were MRD positive post induction with tandem ASCT vs. single ASCT plus 2 cycles of isatuximab, carfilzomib, lenalidomide and dexamethasone (Isa-KRd). There was no significant difference in pre-maintenance MRD negativity rates (10^-6^), suggesting that alternate escalation strategies may be required (108). In patients who were MRD negative post-induction, there was also no significant difference in pre-maintenance MRD negativity rates when treatment was de-escalated to compare ASCT plus 2 cycles of Isa-KRd vs. 6 cycles of Isa-KRd only, suggesting a diminished role of ASCT in MRD negative patients if adequate therapy is administered. Sustained MRD negativity rates and survival data follow-up are ongoing.

There are several in progress studies assessing escalation and de-escalation of therapy tailored to MRD status. The ADVANCE study, will assess patients for MRD (10^-6^) after 8 cycles of either Dara-KRd or KRd and only MRD positive patients will undergo ASCT (109). In the multi-arm RADAR study of NDTE patients receiving bortezomib, cyclophosphamide and dexamethasone (VCD) followed by ASCT, patients who test MRD positive post-ASCT will be randomized to receive either lenalidomide maintenance, VRd consolidation followed by lenalidomide maintenance or isatuximab, bortezomib, lenalidomide and dexamethasone (Isa-VRd) consolidation followed by isatuximab and lenalidomide (Isa-R) maintenance (110). The PERSEUS trial is assessing re-escalation of therapy at MRD resurgence. In this study, patients receiving daratumumab and lenalidomide (Dara-R) maintenance who are MRD negative (10^–5^) after 2 years of maintenance are de-escalated to single agent lenalidomide. In the event of loss of CR or MRD resurgence, they will be re-escalated to Dara-R (1). Follow up is ongoing. **Tables 4** and **5** summarize key published and ongoing MRD adapted trials.

**Table 4:** Published MRD adapted trials.

Study and population	Study design	MRD method and sensitivity	MRD adapted strategy	Outcome
GEM2014MAIN (91) NDTE	Phase III, randomized, open-label trial	NGF at 2×10^–6^ sensitivity	**Discontinuation of maintenance** IRd or Rd after 24 cycles if MRD-	4-year PFS from maintenance discontinuation if MRD- was 82.8% vs. 50.4% with MRD+ and treatment continuation
MASTER (82) NDTE	Phase II, single-arm trial	NGS at 10^–5^ sensitivity	**Discontinuation of treatment** at any time point after 2 consecutive MRD- results	24-month PFS from cessation of therapy was 88%, 85% and 53% for 0, 1 and ≥2 HRCA
MRD2STOP (90) NDTE and NDTI	Prospective, single-institution trial	PET, MFC at 10^–5^ sensitivity and NGS at 10^–6^ sensitivity	**Discontinuation of maintenance** in patients who had received ≥ 12 months maintenance and who were previously known to be MRD- by all 3 modalities	3-year PFS following discontinuation of 85% for all patients; 92% for those MRD- at 10^–7^ at baseline vs. 49% for those MRD+ at 10^–7^ at baseline
PERSEUS (1) NDTE	Phase III, randomized, open-label trial	NGS at 10^–5^ sensitivity	**De-escalation of maintenance** Dara-R to R after 24 months if in CR with 12 months sustained MRD-. Recommenced on loss of CR without disease progression or MRD resurgence	207/322 patients achieved sustained MRD- and discontinued dara. Long-term follow-up is ongoing
MIDAS (108) NDTE	Phase III, randomized, open-label trial	NGS at 10^–5^ sensitivity at randomization and NGS at 10^–6^ sensitivity at primary end point	**Intensification of treatment** by MRD status. Post induction, MRD- randomized to 6x Isa-KRd vs. ASCT and 2× Isa-KRd; MRD+ randomized to single ASCT and 2x Isa-KRd vs. tandem ASCT	Post-induction MRD- group: No significant difference in MRD- at 10^–6^ pre-maintenance for Isa-KRd vs. ASCT (OR 1.17, 95% CI 0.64-2.76, p=0.64) Post-induction MRD+ group: No significant difference in MRD- at 10^–6^ pre-maintenance for single vs. tandem ASCT (OR 0.73, 95% CI 0.42-1.25, p=0.31) Long-term follow-up for sustained MRD negativity and survival data is ongoing
AURIGA (107) NDTE	Phase III, randomized, open-label trial	NGS at 10^–5^ sensitivity	**Compared intensification of maintenance** in MRD+ patients. Randomized to receive Dara-R vs. R	MRD+ to MRD- conversion was 60.6% in the Dara-R group vs. 27.7% in the R group (OR 4.12, 95% CI 2.26-7.52, P<0.0001)
Terpos et al. (89) NDTE	Prospective, single-institution trial	NGF at 2×10^–6^ sensitivity	**Discontinuation of maintenance** R after 3 years of sustained MRD-	3-year PFS from maintenance discontinuation was 92.9%
ATLAS (105) NDTE	Phase III, randomized, open-label superiority trial	MFC or NGS at 10^–5^ sensitivity	**De-escalation of maintenance** KRd to R at cycle 8 if standard-risk cytogenetics and MRD- after 6 cycles. This was compared to receiving single-agent R from cycle 1	4-year PFS from start of maintenance was 67.5% in KRd group even with de-escalation of treatment vs. 38.0% in the R group

MRD, minimal residual disease; NDTE, newly diagnosed transplant eligible; NGF, next generation flow cytometry; IRd, ixazomib, lenalidomide, dexamethasone; Rd, lenalidomide, dexamethasone; PFS, progression free survival; NGS, next generation sequencing; HRCA, high-risk cytogenetic abnormalities; NDTI, newly diagnosed transplant ineligible; PET, positron emission tomography; MFC, multiparameter flow cytometry; R, lenalidomide; Dara, daratumumab; CR, complete response; ASCT, autologous stem cell transplant; Isa-KRd, isatuximab, carfilzomib, lenalidomide, dexamethasone; OR, odds ratio; CI, confidence interval. Bold text specifies key strategy used.

**Table 5:** In progress MRD adapted trials.

Study	Study design	MRD method and sensitivity	MRD adapted strategy
DRAMMATIC/SWOG (104) NDTE	Phase III, randomized, open-label study	NGS	**Discontinuation vs. continuation of maintenance** at 2 years if MRD-
MASTER2 (111) NDTE	Phase II, randomized, open-label study	NGS	**Response-adapted randomization based on MRD status** following induction: MRD-: 3x Dara-VRd intensification, 13× Dara-R maintenance vs. ASCT intensification, 13x Dara-R maintenance MRD+: ASCT intensification, 3x Dara-Tec consolidation, 13x Dara-Tec maintenance vs. ASCT intensification, 3x Dara-R consolidation, 13x Dara-R maintenance Post-maintenance phase patients with sustained MRD- will enter treatment-free observation vs. ongoing R maintenance
REMNANT (112) NDTE	Phase II/III, randomized, open-label study	NGF at 10^–5^ sensitivity	**Initiation of second-line therapy at MRD resurgence vs. PD** during maintenance
RADAR (113) NDTE	Phase II/III, randomized, open-label study	MFC at 10^–5^ sensitivity	**Risk-adapted and response-guided protocol based on MRD status and cytogenetic risk** Standard-risk and MRD+ post ASCT randomized to test treatment intensification with consolidation and maintenance Standard-risk with sustained MRD- after 12 months of maintenance randomized to stop vs. continue maintenance High-risk undergo intensified induction, consolidation, and maintenance
ADVANCE (109) NDTE	Randomized, open-label study	NGS at 10^–5^ sensitivity	**ASCT if MRD+** following induction with 8 cycles of Dara-KRd vs. KRd

MRD, minimal residual disease; NDTE, newly diagnosed transplant eligible; NGS, next generation sequencing; Dara-VRd, daratumumab, bortezomib, lenalidomide, dexamethasone; Dara-R, daratumumab, lenalidomide; ASCT, autologous stem cell transplant; Dara-Tec, daratumumab, teclistamab; NGF, next generation flow cytometry; PD, progressive disease; MFC, multiparameter flow cytometry. Bold text specifies key strategy used.

## 8 The role of MRD in newly diagnosed transplant ineligible multiple myeloma patients

Common MRD assessment landmarks used in NDTI patients include at VGPR, suspected CR/sCR or after a fixed duration (6–12 months) (24). Given the lower response rates to treatment and shorter PFS in these groups of patients, sustained MRD negativity rates are generally low at <20%, leading to a paucity of MRD data in this group of patients (114). In a pooled analysis of two daratumumab-based frontline studies on NDTI patients, MRD negativity predicted for superior PFS rates with sustained MRD negativity achieving the best clinical outcomes (74). With the use of quadruplet therapy in NDTI patients, overall response rates are now approaching 70-90% with VGPR/CR rates exceeding 50% (115). The recently reported CEPHEUS and BENEFIT studies have also shown MRD negativity rates (<10^-5^) in the quadruplet arms (involving a CD38 monoclonal antibody) at 50-60% (115, 116). As such, MRD testing for response assessment has gained greater relevance in these groups as it may help with treatment continuation or de-escalation. This was demonstrated in a phase 2 study of elotuzumab-KRd in patients without transplant intent which allowed cessation of Carfilzomib if patients were MRD negative at <10^–6^ at cycles 8 and 12. Patients who de-escalated treatment at cycle 8 achieved a 3-year PFS of 92% (117). In another phase 2 study of NDMM patients receiving 8 cycles of Dara-KRd induction, patients who were MRD negative at <10^–5^ were de-escalated to lenalidomide maintenance, achieving a sustained MRD negativity rate of 78% for more than 12 cycles (105). Conversely, the MRD positive NDTI group received further KRd consolidation, although none converted to MRD negativity.

## 9 The role of MRD in relapsed refractory myeloma and the era of T-cell redirected therapy

Given the increasing incidence of treatment-resistant disease, MRD negativity rates have been low in the relapsed/refractory cohort of patients receiving standard of care triplet regimens (118). However, the interest in MRD negativity has been growing with the introduction of T-cell redirection therapy. In the CAR-T space, work has been done to characterize the role of MRD testing, particularly its impact on survival and discerning testing timepoints for MRD. Bansal et al. looked at the impact of BM MRD status 1 month after a BCMA-CAR-T (Ide-cel) in RRMM patients. In this group 78% of patients were MRD negative at 1 month but only 40% ultimately achieved sustained MRD negativity (119). Regardless, MRD negativity at 1 month was prognostic, associated with superior PFS (when compared to MRD positivity), demonstrating that early evaluation of MRD post CAR-T may be prognostic (119). Paiva et al. in their longitudinal investigation of patients receiving Ide-cel confirmed similar PFS superiority in achieving MRD negativity, particularly sustained MRD negativity (120). It was also noted that the peak rates of CR (by PB evaluation) lagged behind the peak rates of BM MRD negativity by 3 months likely due to the long half-life of immunoglobulins and suggests that early MRD negativity better reflects the dynamics of CAR-T peak expansion and depth of initial tumour clearance. Recently, an updated analysis of the CARTITUDE-4 study of lenalidomide refractory patients that compared the use of cilta-cel with standard of care triplet therapy has been published showed that 52% in the cilta-cel group achieved sustained MRD negativity of ≥12 months, compared to 10% in the standard of care arm which translated to a superior PFS (121). Conversely, a recent real-world study of cilta-cel treated patients found that BM MRD positivity at 3 months, was associated with inferior PFS and increased risk of early treatment failure (122). Together, these data underscore that in the CAR-T context, both the depth of initial MRD clearance and its sustained maintenance are independently meaningful, with early MRD positivity identifying a high-risk group requiring close monitoring and potentially earlier therapeutic intervention, while durable MRD negativity appears to be the benchmark for optimal long-term disease control.

Considering T-cell redirection therapy, Teclistamab is a humanised BCMA×CD3 bispecific antibody approved to treat relapsed refractory Myeloma and has achieved durable responses exceeding 12 months (5). A subsequent updated analysis has also shown high overall MRD negative, CR rates of 46% in evaluable patients (123). However, infection risk remains a significant concern with extended treatment, which provides impetus for the investigation of fixed duration therapy. The phase II LimiTec trial is an ongoing single-arm non-inferiority study where patients who have received 6–9 months of Teclistamab in VGPR or better can cease therapy. The interim analysis has found that cessation yields FFS (failure-free survival) outcomes comparable to that of continuous treatment in the MajesTEC-1 trial (124). There will be an exploratory component on MRD status in the final analysis.

## 10 Defining “cure” in multiple myeloma

In February 2026 the Myeloma Cure Summit was held in Miami, Florida, USA by the International Myeloma Society (IMS) to discuss what might constitute the definition of cure in MM. The proposed consensus definition of cure was MRD negative (10^-6^) patients who have been off anti-myeloma therapy for 5 years. This definition has not yet been formally published but reflects the prognostic value of sustained MRD negativity and the shift towards MRD-adapted therapy.

## 11 Conclusions

There is sound evidence that MRD negativity is a suitable surrogate endpoint that strongly correlates with survival outcomes regardless of treatment. This has and will continue to heavily impact the conduct of clinical trials, allowing for shortened trial read out times which in turn can be used by regulatory bodies for accelerated drug approval and patient access to effective therapy. MRD testing is increasingly being used for treatment decision-making, such as treatment de-escalation or re-escalation and tailoring treatment intensity based on patient response, which may ultimately reduce toxicity and maximize drug efficacy. There is current good *post-hoc* and phase 2 data that an MRD-directed approach is feasible, and there are currently multiple larger prospective randomized studies testing this concept. With more MRD data and improving testing technology, future directions include assessing the impact of MRD responses at deeper thresholds (10^–6^, 10^–7^ etc.) and redefining the meaning of sustained MRD (1 year vs. 2 vs. 3 years), particularly in the high-risk subgroup. PB-based MRD methods show good correlation with BM-based MRD methods and survival outcomes, but these methods are only restricted to a few academic centres, have variable sensitivities, and are yet to have an agreed predefined threshold of significance. With its non-invasive nature, greater efforts should be made in peripheral blood testing to prove that it is a good surrogate or complement to BM MRD methods, allowing for more frequent and dynamic MRD testing.
